# Isolation of Single-Domain Antibody Fragments That Preferentially Detect Intact (146S) Particles of Foot-and-Mouth Disease Virus for Use in Vaccine Quality Control

**DOI:** 10.3389/fimmu.2017.00960

**Published:** 2017-08-17

**Authors:** Michiel M. Harmsen, Julian Seago, Eva Perez, Bryan Charleston, Phaedra L. Eblé, Aldo Dekker

**Affiliations:** ^1^Wageningen Bioveterinary Research, Lelystad, Netherlands; ^2^The Pirbright Institute, Woking, United Kingdom

**Keywords:** enzyme-linked immunosorbent assay, single-domain antibody, foot-and-mouth disease, virion stability, foot-and-mouth disease virion, vaccine quality control

## Abstract

Intact (146S) foot-and-mouth disease virus (FMDVs) can dissociate into specific (12S) viral capsid degradation products. FMD vaccines normally consist of inactivated virions. Vaccine quality is dependent on 146S virus particles rather than 12S particles. We earlier isolated two llama single-domain antibody fragments (VHHs) that specifically recognize 146S particles of FMDV strain O_1_ Manisa and shown their potential use in quality control of FMD vaccines during manufacturing. These 146S-specific VHHs were specific for particular O serotype strains and did not bind strains from other FMDV serotypes. Here, we describe the isolation of 146S-specific VHHs against FMDV SAT2 and Asia 1 strains by phage display selection from llama immune libraries. VHHs that bind both 12S and 146S particles were readily isolated but VHHs that bind specifically to 146S particles could only be isolated by phage display selection using prior depletion for 12S particles. We obtained one 146S-specific VHH—M332F—that binds to strain Asia 1 Shamir and several VHHs that preferentially bind 146S particles of SAT2 strain SAU/2/00, from which we selected VHH M379F for further characterization. Both M332F and M379F did not bind FMDV strains from other serotypes. In a sandwich enzyme-linked immunosorbent assay (ELISA) employing unlabeled and biotinylated versions of the same VHH M332F showed high specificity for 146S particles but M379F showed lower 146S-specificity with some cross-reaction with 12S particles. These ELISAs could detect 146S particle concentrations as low as 2.3–4.6 µg/l. They can be used for FMD vaccine quality control and research and development, for example, to identify virion stabilizing excipients.

## Introduction

Foot-and-mouth disease (FMD) is an animal disease that is caused by a picornavirus, FMD virus (FMDV), which encompasses seven serotypes: A, O, C, Asia1, SAT1, SAT2, and SAT3. Infection with any one serotype does not produce significant humoral immunity against other serotypes. In FMD endemic areas vaccination is used as a preventive method (1). Due to differences in serotype prevalence in the field most vaccines are used for serotypes O and A. Further vaccines generally are specific for Asia1 or SAT2 serotypes. Conventional FMD vaccines (2) are based on chemically inactivated FMDVs that are formulated with an adjuvant. FMD virions consist of an RNA molecule and a capsid composed of 60 copies each of VP1, VP2, VP3, and VP4 proteins (3). Intact virions sediment at 146S in sucrose gradients. Some FMDV strains also produce empty capsids that lack the RNA molecule and sediment at 75S. Mild heating or incubation at pH below 6.5 leads to irreversible dissociation of 146S or 75S particles into stable 12S particles that contain five copies each of VP1, VP2, and VP3. Dissociation into 12S particles results in a strongly reduced immunogenicity (4–7).

Several methods have been developed to measure the concentration of 146S particles of the crude FMDV antigen preparation used for vaccine preparation. This is traditionally measured by sucrose density gradient (SDG) centrifugation (8). Novel methods that are more easy to automate are based on size-exclusion high-performance liquid chromatography (9, 10) or lateral flow immunoassay (11). All these methods have the advantage of being suitable for all FMDV strains, but the disadvantage of low sensitivity, limited sample throughput, and inability to discriminate different vaccine strains in multivalent vaccines. Double antibody sandwich (DAS) enzyme-linked immunosorbent assays (ELISAs) using monoclonal antibodies (mAbs) were also developed for FMDV antigen quantification (7, 12–16). They are more sensitive, but often not specific for intact 146S particles. Only two DAS ELISAs were specific for 146S particles due to use of a mAb showing such specificity. They were suitable for detection of A or O serotype strains (15, 16). We have recently developed two DAS ELISAs using two recombinant llama single-domain antibody fragments (VHHs) that bind specifically to either 146S particles or 12S particles of strain O_1_ Manisa (17, 18). In each ELISA, the same VHH was used for coating as well as for detection of captured antigen using biotinylated VHH. The DAS ELISA employing 146S-specific VHH M170 was specific for particular O serotype strains, including strain O_1_ Manisa. The DAS ELISA employing 12S-specific VHH M3 could detect FMDV antigen of several A, O, and Asia 1 strains but not SAT2 strain. We were also able to measure 146S particles of A and Asia 1 serotype strains employing the M3 DAS ELISA of heated and untreated samples (17). However, this latter ELISA approach is not suitable for detecting 146S particles in the presence of higher concentrations of 12S particles. An ELISA approach employing a 146S-specific VHH is therefore preferred. We here describe the isolation of two VHHs for specific detection of 146S particles of strains Asia 1 Shamir and SAT2 SAU/2/00.

Many conventional mAbs against FMDV have been isolated in the past. Most of those mAbs bind to both 12S and 146S particles (7, 19–22). However, mAbs that specifically detect 146S particles are rarely isolated (15, 16). VHHs against FMDV have recently been isolated (23, 24) without reporting their particle specificity. M170 is the only 146S-specific VHH that is currently described (17). It was isolated by screening a panel of 24 VHHs against strain O_1_ Manisa that were earlier isolated from llama immune libraries (25). It is not surprising that 146S-specific VHHs or mAbs are rare since 12S and 146S particles share many epitopes.

VHHs are normally isolated by phage display selection from immunized llamas or dromedaries (26). One of the advantages of using VHHs for this purpose is the high functional diversity of immune libraries derived from heavy chain antibodies since random shuffling of antibody heavy and light chains does not occur. Phage display VHH immune libraries therefore contain VHHs with many different specificities. The isolation of rare specificities requires dedicated selection procedures. The removal from libraries of clones with unwanted cross-reaction to particular antigens can be accomplished by prior depletion of libraries on these antigens before performing selection of VHHs on the target antigen (27), although such depletion often is inefficient (28, 29). We here describe the selection of VHHs specific for 146S particles by depletion on 12S particles. We focus on VHHs specific for Asia 1 and SAT2 FMDV since 146S-specific VHHs or mAbs are not yet available for these serotypes.

## Materials and Methods

### mAbs, VHHs, and FMDV Antigens

Monoclonal antibody 13A6 was raised against SAT1 Zimbabwe/89 that does not neutralize FMDV *in vitro*, cross-reacts to SAT2 Zimbabwe/86 and binds in a trypsin-sensitive manner in ELISA (30). VHHs were produced in baker’s yeast as a fusion to the natural llama heavy-chain antibody long hinge region and a hexahistidine tag using vector pRL188 (25) and purified from culture supernatant using immobilized-metal affinity chromatography as described earlier. VHHs produced in this manner are indicated by the suffix “F.” At least 20% of the VHH amount produced in this manner is dimerized through the single cysteine present at the C-terminus of the VHH, immediately preceding the his6 tag. Both such monomeric and dimeric VHHs are useful for functional immobilization of VHHs to polystyrene surfaces by passive adsorption (31). Yeast-produced VHHs were biotinylated at a weight ratio of protein to biotin of 10 using amine-reactive sulfo-*N*-hydroxysuccinimide-LC-biotin (Pierce, Rockford, IL, USA). Buffer was exchanged to PBS and free biotin removed by at least three consecutive 10-fold dilutions and concentration using Amicon Ultra 3-kDa molecular weight cutoff concentration devices (Millipore, Billerica, MA, USA). The VHH2 M3ggsVI4_Q6E_ specific for 12S particles has been previously described (32).

The FMD vaccine strains Asia 1 Shamir/Israel/89, SAT2 SAU/2/00, O_1_ Manisa/Turkey/69, O_1_ British Field Strain 1860 (BFS)/67, A Turkey/98, and A_24_ Cruzeiro/Brazil/55 were used for antigen production. FMDV antigen originated from the virus production facilities in Lelystad. FMDV was cultured using BHK-21 cells grown in suspension in industrial size bioreactors. FMDV present in the clarified culture was inactivated with 0.01 M binary ethylenimine and concentrated using two consecutive polyethylene glycol-6000 precipitations, resulting in crude antigen. Infectious FMDV was prepared at laboratory scale by growing them on monolayers of BHK-21 cells and harvesting the culture supernatant without further treatment. Work with infectious FMDV was done in a high containment unit at Wageningen Bioveterinary Research using appropriate measures for virus handling and waste disposal to prevent FMDV introduction into the environment. Wageningen Bioveterinary Research is authorized to work with live FMDV according to EU directive 2003/85/EC.

Foot-and-mouth disease virus antigens were fractionated using 10–40% SDGs that were centrifuged for 2.5 h at 10°C and 200,000 × *g*. The gradients were fractionated into 20 0.61 ml aliquots and the absorbance at 254 nm was determined to identify the 146S peak. The 146S concentration in milligrams per liter was then calculated by multiplying the absorbance at 254 nm with 126.7. 12S particles were prepared from 146S preparations by acidification (12S^A^) or heat treatment for 1 h at 56°C (12S^H^) as previously described (33).

### Llama Immunization and Phage Library Construction

Llama immunizations were performed after ethical review by Wageningen Bioveterinary Research and in accordance with Dutch national guidelines on animal use. The immunization of llama 6058 with crude antigen of FMDV strain Asia 1 Shamir has been described earlier (25). The immunization of llamas 3049 and 3050 with influenza antigens and subsequent phage library construction was earlier described (34). Simultaneously with these influenza antigens llamas 3049 and 3050 were immunized with FMDV 146S particles purified from SDG. For each of the three immunizations llama 3049 received 40 µg Asia 1 Shamir 146S particles and 45 µg A_24_ Cruzeiro 146S particles, whereas llama 3050 received 50 µg SAT2 SAU/2/00 146S particles. Phage display immune libraries in phagemid vector pRL144 (35) of at least 10^7^ independent clones were generated as previously described (25, 34).

### Phage Display Selection of Antigen Binding VHHs

Phage display selections were performed by two consecutive rounds of biopanning (36) in polystyrene 96-well plates (Greiner, Solingen, Germany, Cat. No. 655092), using 100 µl/well for each incubation. Many variations on the procedure were used (Table 1). In general, antigen was immobilized to 96-well plates at three serial 10-fold dilutions and a control without antigen was included. VHHs were normally coated at concentrations of 1, 0.1, and 0.01 mg/l in 0.05 M carbonate/bicarbonate buffer, pH 9.6 (coating buffer), overnight at 4°C. Purified 146S particles (1 mg/l) were separately incubated in PBS buffer containing 1% milk and 0.05% Tween-20 (PBSTM) for 1 h at room temperature (RT). Controls included wells coated with VHH without subsequent incubation with FMDV antigen and uncoated wells incubated with 1 mg/l FMDV antigen. Plates were then incubated with 1 × 10^9^ transducing phage units per well. In initial experiments, phages were preincubated in PBSTM containing 10 mg/l 12S^A^ particles and then added to wells with captured 146S particles to compete for binding to 12S^A^. In later experiments, phages binding to 12S^A^ particles were depleted prior to biopanning by incubation on plates containing immobilized 12S^A^ particles. For this purpose, separate plates were coated with coating buffer containing 1 mg/l M3ggsVI4_Q6E_ or M311F specific for SAT2 SAU/2/00 antigen and subsequently incubated with 5 mg/l 12S^A^ particles of strains Asia 1 Shamir or SAT2 SAU/2/00, respectively. Phages were then incubated on these plates with captured 12S^A^ particles after which the unbound phages were transferred to biopanning plates containing captured 146S particles. To ensure binding of most 12S^A^ reactive phages we used 20-fold lower amounts of phage (5 × 10^7^ transducing phage units per well) when employing depletion. Bound phages were finally eluted by incubation with 1 mg/ml trypsin in PBS for 30 min at 37°C and immediately transduced to *Escherichia coli* TG1 [(F′ *traD36 proAB lacIqZ* Δ*M15*) *supE thi-1* Δ*(lac-proAB)* Δ(mcrB-hsdSM)5(rK^−^*mK*^−^*)*] cells. In each selection round, we performed a phage ELISA simultaneous with the phage display selection for evaluation of the phage display selection. For this purpose, a duplex plate containing similar concentrations and types of antigen and phage was incubated with a peroxidase-conjugated mAb against M13 phage instead of incubation with trypsin. The amount of bound antigen-specific phage was then measured by phage ELISA.

**Table 1:** Phage display conditions used to retrieve FMDV binding VHHs.

Llama	Coating		Captured FMDV		Competing FMDV		Depleting FMDV		VHHs selected	No. VHH sequenced	No. Unique VHHs	No. CDR3 groups
	Conc. (mg/l)	Protein	Conc. (mg/l)	FMDV antigen	Conc. (mg/l)	FMDV Antigen	Conc. (mg/l)	FMDV antigen				
6058	1	Streptavidin	0.1	Biotinylated Asia 1 Shamir antigen	–	None	–	None	M98	1	1	1
3050	0.1	mAb13A6	0.5	SAT2 SAU/2/00 146S	10	SAT2 SAU/2/00 12S^A^	–	None	M311–M317	7	5	4
3050	0.1	M311F	0.5	SAT2 SAU/2/00 146S	–	None	5	SAT2 SAU/2/00 12S^A^	M371–M381	11	7	3
3049	0.1	M98F	0.5	Asia 1 Shamir 146S	10	Asia 1 Shamir 12S^A^	–	None	M301–M308	8	8	7
3049	0.1	M98F	0.5	Asia 1 Shamir 146S	–	None	5	Asia 1 Shamir 12S^A^	M331–M337	7	7	6
6058												

*FMDV, foot-and-mouth disease virus*.

### Production of Soluble VHH in *E. coli*

After the second round of panning phages was transduced to *E. coli* TG1 cells, individual colonies were picked and the VHH genes were induced with 1 mM isopropyl β-d-thiogalactopyranoside. Recombinant VHHs, extracted from the periplasm, were tested for binding to FMDV antigens at 10-fold dilution as described below in Section “Enzyme-Linked Immunosorbent Assays.”

### Sequence Analysis

Sequence analysis of the VHH encoding region was performed, as previously described (37). The deduced VHH amino acid sequences were aligned according to the IMGT system (38) for alignment, numbering, and complementarity-determining region definition of immunoglobulins. VHHs were classified into subfamilies as earlier defined (37). Potential N-glycosylation sites were defined as Asn-X-Ser/Thr, where X represents any amino acid, except Pro.

### Enzyme-Linked Immunosorbent Assays

Three different ELISA procedures were used. We first describe the basic ELISA procedure that was commonly used in these three procedures. ELISAs were performed by coating high-binding polystyrene 96-well plates (Greiner) with 0.1–1 mg/l of unlabeled VHH, VHH2, or mAb in 50 mM carbonate/bicarbonate buffer, pH 9.6 (coating buffer), overnight at 4°C. Coating and subsequent incubations were performed using 100 µl per well. After washing, the coated plates were incubated with FMDV antigens in ELISA-buffer (1% skimmed milk; 0.05% Tween-20; 0.5 M NaCl; 2.7 mM KCl; 2.8 mM KH_2_PO_4_; 8.1 mM Na_2_HPO_4_; pH 7.4) or PBSTM for 1 h at RT. The 146S concentration of the FMDV antigens was determined by measuring the absorbance at 254 nm as described above. The concentration of the 12S antigens was derived from the 146S concentration of the sample from which it was prepared assuming complete conversion of 146S into 12S particles. Plates were next incubated with either phage-displayed VHH, *E. coli*-produced VHH, or yeast-produced biotinylated VHH, and subsequently with a suitable specific peroxidase conjugate, using the same buffer as used for incubation with FMDV antigen (ELISA-buffer or PBSTM). The peroxidase conjugate was subsequently detected by staining with 3,3′,5,5′-tetramethylbenzidine. After stopping the reaction by addition of 0.5 M H_2_SO_4_ (50 µl per well) the absorbance at 450 nm was measured using a Multiskan Ascent spectrophotometer (Thermo Labsystems, Helsinki, Finland).

For phage ELISA plates were preferably coated with low concentrations of VHH (0.1 mg/l) and FMDV antigen (0.5 mg/l) as described in Table 1. Many variations on the procedure were used during phage display selection as described above. Bound phage displayed VHH was detected by incubation with peroxidase-conjugated mAb against the M13 p8 coat protein (GE Healthcare, Little Chalfont, UK) in PBSTM.

For ELISA using *E. coli*-produced soluble VHH plates were preferably coated with 1 mg/l VHH and then subsequently incubated with 1 mg/l FMDV antigen in ELISA buffer. Bound FMDV antigens were detected by incubation with 10-fold diluted *E. coli*-produced soluble VHH, which contains a myc-tag, and a peroxidase-conjugated mAb against the c-myc tag (clone 9E10; Roche Applied Science).

For DAS ELISA to measure the FMDV antigen concentration plates were coated with 0.5 mg/l of unlabeled VHH or VHH2. These plates were then incubated with serial 2-fold dilution series of FMDV antigen preparations in ELISA buffer. Normally, standards of untreated FMDV antigen were included in the ELISAs for quantification of 146S particles. However, 12S^A^ preparations of FMDV antigen were used as standards in the ELISA employing M3ggsVI-4_Q6E_. Standards were serial twofold dilutions of 1 mg/l start concentration. Plates were next incubated with 0.25 mg/l of biotinylated VHH(2). Bound biotinylated VHH(2) was detected with 1 mg/l PO-conjugated streptavidin (Jackson ImmunoResearch Laboratories Inc., West Grove, PA, USA). Absorbance data were evaluated using an Excel^®^ spreadsheet template (Microsoft Corporation, Redmond, WA, USA). A four-parameter logistic curve was fitted to absorbance and FMDV antigen concentrations of standards by non-linear least squares using the Excel^®^ solver tool. The FMDV antigen concentration in unknown samples was then determined by interpolation. For determination of the quality of binding of VHHs to particular FMDV antigens these antigens were titrated in twofold dilution series. The effective antigen concentration required to reach a particular absorbance value was then interpolated after four-parameter logistic curve fitting of absorbance and antigen concentrations.

The limit of detection (LOD) of ELISAs was measured by titrating FMDV antigens in triplicate and interpolating the VHH concentration required to reach the average and three times the SD of nine blank measurements without antigen.

## Results

### Selection of 146S-Specific VHHs

We earlier immunized llama 6058 with crude antigen of FMDV strains O_1_ Manisa, A Turkey, A_22_ Iraq, and Asia 1 Shamir. We isolated many FMDV O_1_ Manisa binding VHHs from this llama by phage display employing biotinylated FMDV antigen captured with streptavidin-coated magnetic beads (25). The VHH M98 was similarly selected from llama 6058 using biotinylated FMDV Asia 1 Shamir antigen. Yeast-produced M98F VHH neutralized Asia 1 Shamir FMDV *in vitro*, confirming M98F binds FMDV virions rather than a host protein present in the crude FMDV antigen used to perform the selection. As further confirmed below M98F was found to bind both 12S and 146S particles. This was not unexpected since 146S-specific VHHs were also rarely retrieved during phage display selection of O_1_ Manisa binding VHHs (25).

To improve the selection process of 146S-specific VHHs we purified 146S particles by SDGs. The purified 146S particles were then used for both novel llama immunization and phage display selection of VHHs. One llama (3049) was immunized with FMDV Asia 1 Shamir 146S and another llama (3050) with SAT2 SAU/2/00 146S. As observed with many other proteins (39) FMDV loses much of its antigenicity upon direct passive adsorption to polystyrene (40). We, therefore, used M98F VHH that binds Asia 1 Shamir and mAb 13A6 that binds SAT2 SAU/2/00 to capture purified 146S particles in ELISA. We initially tried to select VHHs that bind specifically to 146S particles by competition during phage display selection with a surplus of 12S^A^ particles. This approach was not successful as all VHHs isolated bound both 146S and 12S^A^ particles when tested as soluble VHH produced from *E. coli* TG1 cells (results not shown). After sequence analysis (Table 1), we identified five unique clones (M311-M317) binding SAT2 SAU/2/00 and eight unique clones (M301–M308) binding Asia 1 Shamir 146S. Their cross-reaction with 12S was later confirmed using yeast-produced VHH (Table 2). To increase enrichment of 146S-specific VHHs we improved phage display selections in two ways. Since mAb 13A6 preferentially binds 12S^A^ particles (see below), it was replaced by a novel isolated VHH that was highly produced in yeast, M311F, for capture of SAT2 SAU/2/00 146S particles. Furthermore, novel selections were performed using depletion with immobilized 12S^A^ particles instead of competition with soluble 12S^A^ particles. Using these novel selection procedures we retrieved seven unique SAT2 SAU/2/00 binding VHHs comprising three novel CDR3 groups (Table 1) that are different from earlier isolated SAT2 SAU/2/00 binding VHHs (Table 2) and seven unique Asia 1 Shamir binding VHHs comprising six CDR3 groups (Table 1) that are mostly different from earlier isolated VHHs (Table 2).

**Table 2:** VHH sequence characteristics and specificity of VHHs for 12S and 146S particles in DAS ELISA using the same VHH for coating and as biotinylated VHH.

VHH or mAb	Llama	CDR3 sequence	VHH subfamily^a^	Antigen concentration (μg/l) to reach absorbance = 0.4			Ratio 12S^H^/146S
				antigen	146S	12S^H^	
**SAT2 SAU/2/00**							
M311F	3050	NAITYYTDAPDY	2	2.7	2.2	5.7	2.6
M314F	3050	AADKWLYISGWRHCRPVFGS	3	8.3	20	17	0.85
M315F	3050	YGDIRVRNY	2	5.7	3.6	17	4.7
M317F	3050	AADWRFVEAVAGRAKY	3	27	66	95	1.4
M377F	3050	NALVLSSSWSEGDY	2	0.76	0.58	5.9	10
M379F	3050	NLVNWGYGENY	2	1.8	1.2	34	28
M380F	3050	NYQRPLSNDNY	2	0.70	0.59	8.7	15
mAb 13A6	NA^b^	NA	NA	>1,000^c^	>1,000	164	<0.16
**Asia 1 Shamir**							
M98F	6058	AAQSPGMSGTYSRSDVYPY	1	4.1	5.5	29.8	5.4
M301F	3049	AATEDYYSGSLGSYYVCPDYYNMDY	3	60	67	>1,000	>15
M303F	3049	AADEPERVYCRDYVRTQYPMDY	3	53	81	>1,000	>12
M304F	3049	AADPPDQDYCSDYDVTVGTELWGS	3	35	42	>1,000	>24
M306F	3049	AADQGAYCSDHGEIGYYGMDY	3	45	62	>1,000	>16
M307F	3049	AAAPEDYYCSDYDGPSEDDYGMDY	3	38	51	>1,000	>20
M308F	3049	AGDQDPAYCSDYWDLNEYDY	3	39	55	>1,000	>18
M332F	3049	AAAWSFRSDYGARLKSAYDF	1	8.4	7.6	433	57
M334F	3049	AADDSGLHGCSDYWILYEYEY	3	245	342	>1,000	>2.9
M336F	6058	AAEESWSTSTYYYTHSYSY	1	>1,000	>1,000	>1,000	ND
M3ggsVI4_Q6E_	NA	NA	NA	51	746	13	0.017

*^a^As defined by reference (37)*.

*^b^NA, Not applicable*.

*^c^>1,000 indicates absorbance does not exceed 0.4 at the highest VHH concentration analyzed (1 mg/l)*.

*DAS, double antibody sandwich; ELISAs, enzyme-linked immunosorbent assays; mAb, monoclonal antibody*.

### 146S Specificity of VHHs

Seventeen VHHs with different CDR3 sequences (Table 2) were produced by yeast expression. They all lacked potential *N-*glycosylation sites to prevent compromised antigen binding due to fortuitous *N*-glycosylation. The purified VHHs were biotinylated for use in DAS ELISA in combination with the same unlabeled VHH for coating. To assess the specificity of the biotinylated VHHs for the different FMDV particles we performed DAS-ELISAs with a titration series of untreated antigen, 146S particles and 12S^H^. Similar to earlier methods (17), the FMDV antigen concentration required to reach an absorbance value of 0.4 was calculated for each FMDV antigen. The absorbance value of 0.4 was chosen because for all different ELISAs used it is sufficiently above background absorbance values to be indicative of antigen binding and sufficiently below the maximal absorbance value. The ratio between these antigen concentration values for 12S^H^ and 146S was taken as a measure for particle specificity (Table 2). A ratio > 1 corresponds to 146S specificity and a ratio < 1 corresponds to 12S specificity.

In the case of SAT2 SAU/2/00 antigen mAb 13A6 shows 12S specificity with a ratio below 0.16, the four VHHs selected by competition with 12S^A^ show no or limited 146S specificity with ratios of 0.85–4.7, whereas the three VHHs selected by depletion with 12S^A^ (M377F, M379F and M380F) show higher 146S specificity with ratios of 10–28 (Table 2). M379F exhibited the highest 146S specificity and recognized relatively low antigen and 146S concentrations as compared to other SAT2 SAU/2/00 binding VHHs. Among the Asia 1 Shamir binding VHHs, M332F that was selected by depletion on 12S^A^ particles showed highest 146S specificity with a ratio of 57. Furthermore, M332F recognized lower concentrations of untreated antigen and 146S as compared to most of the other nine VHHs. Only M311F recognized slightly lower antigen concentrations, although with far lower 146S specificity (Table 2). In conclusion, M332F exhibited high 146S specificity for strain Asia 1 Shamir, whereas M379F showed some 146S specificity for strain SAT2 SAU/2/00.

**Figure 1:**
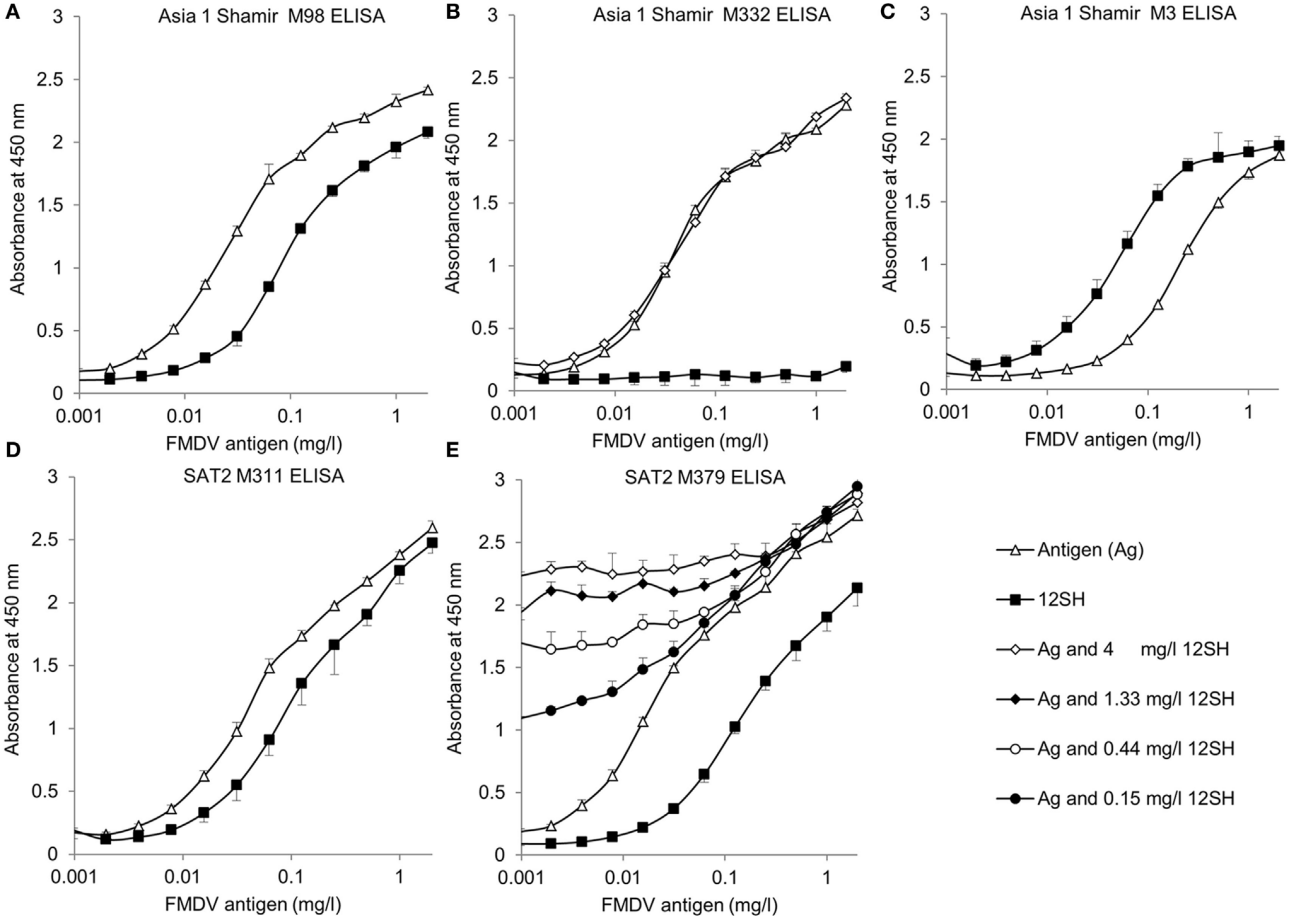
Titration of foot-and-mouth disease virus (FMDV) antigens or 12S^H^ particles derived thereof in double antibody sandwich (DAS) enzyme-linked immunosorbent assays (ELISAs). FMDV strains Asia 1 Shamir **(A–C)** and SAT2 SAU/2/00 **(D,E)** were used. The different DAS ELISAs employed the same VHH for coating in unlabeled form and for detection in biotinylated form. The different VHHs used are indicated above the panels. Antigen or 12S^H^ particles were twofold titrated with a starting concentration of 2 mg/l. The two ELISAs using 146S-specific VHHs **(B,E)** were also titrated with a 2-fold dilution series of antigen to which a constant amount of 12S^H^ particles was added. In the legend the concentration of the constant amount of 12S^H^ particle is indicated. ELISAs were performed in triplicate. Mean and SD are indicated.

We next titrated FMDV antigens and 12S^H^ of strains SAT2 SAU/2/00 and Asia 1 Shamir in triplicate in the M3ggsVI4_Q6E_, M311F, M379F, M98F, and M332F ELISAs using the same VHH as used for detection also for coating. The LOD was calculated for each ELISA (Table 3). The M332F ELISA had an LOD of 4.6 µg/l and the M379F ELISA of 2.3 µg/l for antigen. We also titrated the antigens in these latter two ELISAs in the presence of a constant amount of 12S^H^ particles to determine if antigen quantification is affected by the presence of 12S^H^ particles. Asia 1 Shamir antigen quantification in the M332F ELISA was not affected by the presence of 4 mg/l 12S^H^ (Figure 1B) but SAT2 SAU/2/00 quantification in the M379F ELISA was affected already by the lowest 12S^H^ concentration analyzed (0.15 mg/l; Figure 1E). This is consistent with the earlier observed higher specificity of M332F for 146S particles than M379F.

**Table 3:** LOD of different DAS ELISAs.

FMDV strain	DAS ELISA	LOD (μg/l)	
		Antigen	12S^H^
SAT2 SAU/2/00	M311F	5.2	12
SAT2 SAU/2/00	M379F	2.3	19
Asia 1 Shamir	M98F	3.8	19
Asia 1 Shamir	M332F	4.6	>2,000^a^
Asia 1 Shamir	M3ggsVI4_Q6E_	17	83

*^a^>2,000 indicates LOD not reached at the highest VHH concentration used (2 mg/l)*.

*DAS, double antibody sandwich; ELISAs, enzyme-linked immunosorbent assays; LOD, limit of detection*.

### FMDV Strain Specificity

To further characterize the 17 VHHs, their respective ability to bind to strains of other FMDV serotypes was investigated. As a control we included mAb 13A6 and M8ggsVI4_Q6E_, to demonstrate successful immobilization of the various antigens (Table 4). All seven SAT2 SAU/2/00 binding VHHs did not cross-react to strains Asia 1 Shamir, A Turkey, A_24_ Cruzeiro, O_1_ BFS, or O_1_ Manisa. Similarly, all 10 Asia 1 Shamir binding VHHs did not cross-react to strains SAT2 SAU/2/00, A Turkey, A_24_ Cruzeiro, O_1_ BFS, or O_1_ Manisa (Table 4).

**Table 4:** Binding of biotinylated VHHs to different FMDV strains.

Biotinylated VHH(2) or mAb	VHH concentration (μg/l) to reach absorbance of 0.4					
	SAT2 SAU/2/00^a^	Asia 1 Shamir^b^	A Turkey^c^	A_24_ Cruzeiro^c^	O_1_ BFS^d^	O_1_ Manisa^d^
M311F	0.25	>1,000^e^	>1,000	>1,000	>1,000	>1,000
M314F	2.8	>1,000	>1,000	>1,000	>1,000	>1,000
M315F	0.27	>1,000	>1,000	>1,000	>1,000	>1,000
M317F	0.06	>1,000	>1,000	>1,000	>1,000	>1,000
M377F	1.6	>1,000	>1,000	>1,000	>1,000	>1,000
M379F	0.29	>1,000	>1,000	>1,000	>1,000	>1,000
M380F	1.6	>1,000	>1,000	>1,000	>1,000	>1,000
mAb 13A6	39	21	11	12	566	450
M98F	>1,000	2.5	>1,000	>1,000	>1,000	>1,000
M301F	>1,000	0.59	>1,000	>1,000	>1,000	>1,000
M303F	>1,000	0.14	>1,000	>1,000	>1,000	>1,000
M304F	>1,000	0.50	>1,000	>1,000	>1,000	>1,000
M306F	>1,000	1.8	>1,000	>1,000	>1,000	>1,000
M307F	>1,000	0.75	>1,000	>1,000	>1,000	>1,000
M308F	>1,000	0.11	>1,000	>1,000	>1,000	>1,000
M332F	>1,000	5.4	>1,000	>1,000	>1,000	>1,000
M334F	>1,000	2.0	>1,000	>1,000	>1,000	>1,000
M336F	>1,000	213	>1,000	>1,000	>1,000	>1,000
M8ggsVI4_Q6E_	>1,000	284	113	398	66	5.2

*^a^1 mg/l FMDV antigen captured with 1 mg/l M311F*.

*^b^1 mg/l FMDV antigen captured with 1 mg/l M98F*.

*^c^1 mg/l FMDV antigen captured with 1 mg/l M8F*.

*^d^1 mg/l FMDV antigen captured with 1 mg/l M170F*.

*^e^>1,000, absorbance does not exceed 0.4 at the highest VHH concentration used (1 mg/l)*.

*BFS, British Field Strain 1860; FMDV, foot-and-mouth disease virus; mAb, monoclonal antibody*.

### Binding to 12S, 75S, and 146S Particles in SDGs

Next, we analyzed the specificity of M332F and M379F, as well as the earlier isolated M170F, for different FMDV particles (12S, 75S, and 146S) present in SDG fractions. As controls, we also analyzed each fraction using VHHs that detect both 12S and 146S particles of Asia 1 Shamir, SAT2 SAU/2/00, or O_1_ Manisa.

**Figure 2:**
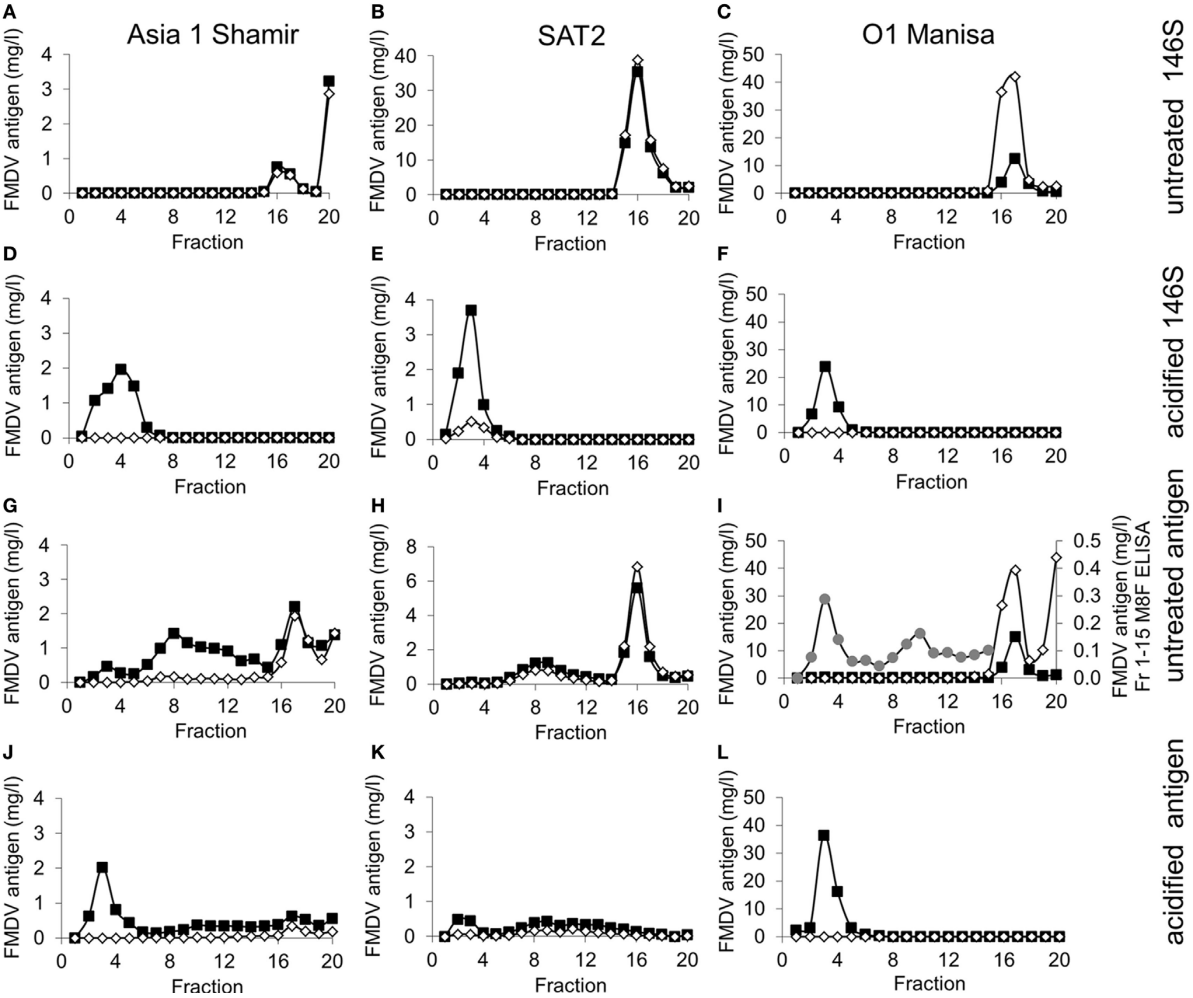
Specificity of double antibody sandwich (DAS) enzyme-linked immunosorbent assays (ELISAs) for inactivated foot-and-mouth disease virus (FMDV) antigen fractionated by sucrose density gradient (SDG). FMDV strains Asia 1 Shamir **(A,D,G,J)**, SAT2 SAU/2/00 **(B,E,H,K)**, and O_1_ Manisa **(C,F,I,L)** were used. FMDV 146S particles purified by SDG were again layered on SDG without further treatment **(A–C)** or after prior acidification for conversion into 12S^A^ particles **(D–F)**. Crude FMDV antigen was similarly fractionated on SDG without further treatment **(G–I)** or after prior acidification **(J–L)**. Twenty fractions of each SDG were analyzed by ELISA using either a VHH that is specific for 146S particles (open diamonds) or that binds both 12S and 146S particles (closed squares). The different 146S-specific VHHs used were M332F **(A,D,G,J)**, M379F **(B,E,H,K)**, and M170F **(C,F,I,L)**. The different 12S- and 146S-recognizing VHHs used were M98F **(A,D,G,J)**, M311F **(B,E,H,K)**, and M8F **(C,F,I,L)**. In panel **(I)**, the low amount of FMDV antigen detected in fractions 1–15 by M8 ELISA is visualized by plotting on a different scale (right axis; gray circles). FMDV antigen concentrations in fractions were calculated from titration series in ELISA against a standard of untreated FMDV antigen with known 146S content. Fraction 1 corresponds to top of gradient.

When FMDV Asia 1 Shamir, SAT2 SAU/2/00, or O_1_ Manisa purified 146S particles were again separated on SDG a peak was seen in fractions 15–18 that corresponds to 146S particles (Figures 2A–C). The absence of further peaks indicated virions had not dissociated into smaller particles during virus handling. The peak in fraction 20 of Asia 1 Shamir FMDV (Figure 2A) probably represents aggregated 146S particles. When 146S particles purified from SDG were acidified and again subjected to SDG only fractions 2–5 contained FMDV antigen (Figures 2D–F), indicating full conversion into 12S^A^ particles. When crude antigen was separated on SDG some antigenic material was detected in fractions 6–14 by ELISA (Figures 2G–I). This could represent 75S particles that sediment between 12S^A^ and 146S particles, although 75S particles would be expected to form a sharper peak. The amount of putative 75S particles as compared to 146S particles is especially high for strain Asia 1 Shamir (Figure 2G), somewhat lower but still considerable for strain SAT2 SAU/2/00 (Figure 2H) and quite low, but clearly detectable for strain O_1_ Manisa (Figure 2I). To visualize the low amount of putative 75S particles of strain O_1_ Manisa the material in fractions 1–15 was plotted on a scale of 0–0.5 mg/l on the right axis (Figure 2I). When crude antigen was acidified prior to SDG fractionation a 12S peak was readily observed for Asia 1 Shamir (Figure 2J) and O_1_ Manisa (Figure 2L), but was less evident for SAT2 SAU/2/00 (Figure 2K). This could have been due to a combination of reduced 12S^A^ particle reactivity in the M311F ELISA and the use of untreated antigen that predominantly consisted of 146S particles as a standard. Acidified O_1_ Manisa antigen consisted only of 12S particles, indicating full dissociation of 146S and 75S particles into 12S (Figure 2L). However, acidified SAT2 SAU/2/00 antigen also contained a broad peak in the fractions corresponding to 75S particles (Figure 2K), whereas Asia 1 Shamir antigen contained both a broad peak corresponding to putative 75S particles as well as a 146S peak, although both peaks were considerably lower as compared to untreated antigen (Figure 2J). This indicates that, unlike SDG purified 146S particles, 75S and 146S particles present in Asia 1 Shamir crude antigen, and 75S particles present in SAT2 SAU/2/00 antigen are only partially converted into 12S by acidification. Such a difference in acid liability between SDG purified and crude antigen could be due to matrix effects due to different buffer composition, as was also reported earlier (41).

The M170F DAS ELISA detects only O_1_ Manisa 146S particles (Figures 2C,F,I,L). The M332F DAS ELISA detects both Asia 1 Shamir 146S and the putative 75S particles, although the latter are detected with reduced efficiency as compared to the M98F DAS ELISA (Figures 2A,D,G,J). The M379F DAS ELISA detects both SAT2 SAU/2/00 146S and putative 75S particles with an efficiency that is equal to the M311F DAS ELISA, whereas SAT2 SAU/2/00 12S^A^ particles are detected with reduced efficiency as compared to the M311F ELISA (Figures 2B,E,H). The binding of M379F to 12S^A^ particles, although with reduced efficiency compared to 146S particles, is consistent with the relatively low 146S specificity observed for M379F.

**Figure 3:**
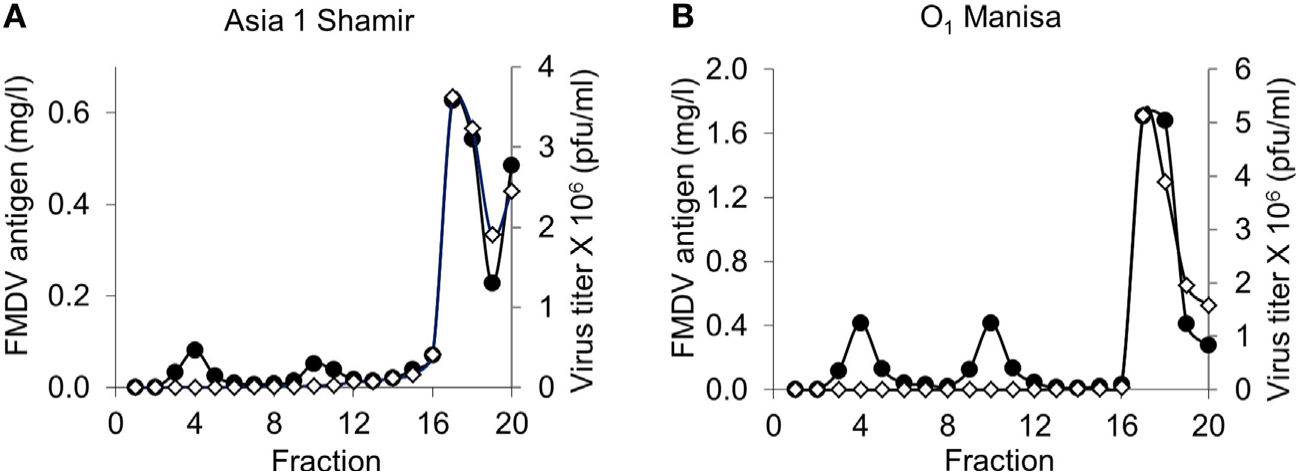
Specificity of double antibody sandwich (DAS) enzyme-linked immunosorbent assays (ELISAs) for infectious foot-and-mouth disease virus (FMDV) particles fractionated by sucrose density gradient (SDG). Infectious virus of FMDV strain Asia 1 Shamir **(A)** or O_1_ Manisa **(B)** was fractionated on SDG. Each fraction was analyzed for infectious FMDV titer (open diamonds) or the FMDV antigen concentration (closed circles). FMDV antigen concentration was measured by M3 ELISA on samples that were first acidified for conversion into 12S^A^ particles against a 12S^A^ standard. Fraction 1 corresponds to top of gradient.

To elucidate the nature of the putative 75S particles we fractionated infectious FMDV on SDG and determined the presence of infectious virus in each fraction by plaque assay. In addition, we determined the concentration of total FMDV antigen present in each fraction by M3 ELISA after acidification. The M3 ELISA facilitates reliable quantification of 12S, 75S, and 146S particles against a 12S standard. This analysis was only done for strains Asia 1 Shamir and O_1_ Manisa since strain SAT2 SAU/2/00 is not detected by M3 ELISA (Figures 3A,B). Notably, FMDV O_1_ Manisa infectious virus contained a considerable putative 75S peak (Figure 3B). Infectious virus was only found in fractions 16–19, consistent with the notion that this represents 146S particles and that FMDV antigen in fractions 9–11 represent 75S particles.

## Discussion

We aimed to isolate VHHs that specifically bind to FMDV 146S particles of strains SAT2 SAU/2/00 and Asia 1 Shamir. For this purpose, llamas 3049 and 3050 were immunized with SDG purified 146S particles of these strains. We preferentially used a 12S depletion step in phage display selections to enrich for 146S-specific VHHs. In this manner, we isolated three novel SAT2 SAU/2/00 binding VHHs showing reasonable 146S specificity and one novel Asia 1 Shamir binding VHH showing high 146S specificity. However, when using a depletion step there is some risk of detachment of captured 12S particles and carryover of this material into the wells containing 146S particles for panning, resulting in binding to capturing VHH and unwanted enrichment for 12S binding phage clones. Since 12S binding VHHs are generally not serotype specific this can be prevented by using 12S particles from another strain for depletion in combination with a serotype-specific VHH for capturing 146S particles. We currently favor this approach for isolation of 146S-specific VHHs. Selection of VHHs specifically recognizing *Taenia solium* antigen without cross-reacting with other *Taenia* species was earlier described (27). Furthermore, VHHs recognizing a recombinant immunotoxin without binding an undesired deamidated derivative of this immunotoxin were isolated recently (42). Together with our study these are further examples where the high functional diversity of camelid heavy-chain antibody immune libraries in combination with careful phage display selection schemes allows isolation of rare VHHs that can discriminate closely related antigens.

We developed two DAS ELISAs that utilize the same yeast-produced VHH for coating, as well as detection when in a biotinylated form, for quantification of FMDV 146S particles. The ELISA with M379F VHH for quantification of SAT2 SAU/2/00 FMDV required 28-fold higher 12S concentrations than 146S concentrations to reach similar absorbance values. Thus, it shows reasonable specificity for 146S particles. However, it cannot be used for quantification of 146S particles in the presence of high concentrations of 12S particles due to cross-reaction with 12S particles. Since the 12S content of vaccines is generally less than 20% of total FMDV antigen (18), such specificity is sufficient to measure the 146S content of SAT2 vaccines with only an about 1% error. Similar analyses performed with M332F VHH to quantify Asia 1 Shamir exhibited a 51-fold increase in specificity for 146S particles, revealing this VHH could be used for 146S quantification in the presence of high concentrations of 12S particles. Both ELISAs showed an LOD of 2.3–4.6 µg/l 146S. Assuming that the antigen concentration in FMDV vaccines is about 10 mg/l and that extraction of antigens from vaccines for use in VHH-ELISA involves a 10-fold dilution of vaccines (18), such sensitivity is amply sufficient for quantification of FMDV antigens in vaccines as well as stability studies of vaccines, which requires measurement of FMDV antigen concentrations that decrease to even lower levels. However, when needed for other applications the LOD could possibly be further increased by genetic fusion of VHH domains to increase affinity as was earlier demonstrated for an antitumor necrosis factor alpha VHH (43) and many further VHHs. In addition, both ELISAs did not detect strains belonging to other serotypes. The M332F ELISA also does not recognize strain Asia 1 Bahrain (results not shown). Both 146S-specific VHHs bind in a serotype-specific manner, and M332F binding is also strain specific. Such coincidence of strain and serotype specificity and 146S specificity was observed before with the 146S-specific O_1_ Manisa binding VHH M170F (25) and two 146S-specific A serotype strain binding mAbs (15, 16). This suggests that the 146S-specific antigenic sites that we now detect on strains of O, SAT2, and Asia 1 serotypes are located on the same region of the FMDV capsid. This notion is consistent with the conservation of three of the four neutralizing antigenic sites between O, A, C, and Asia 1 serotype FMDV strains (44). The 146S-specific antigenic site most likely overlaps with the interface of two 12S pentamers.

Double antibody sandwich enzyme-linked immunosorbent assay analysis of SDG fractionated inactivated FMDV antigen of strains SAT2 SAU/2/00, Asia 1 Shamir, and O_1_ Manisa reveals that some immunoreactive material fractionates between the 12S and 146S peaks (Figure 2). This material could represent 75S empty capsids, although the peak is shallower compared to the 12S and 146S peaks, especially for strain Asia 1 Shamir. Consistent with this notion SDG fractionation of infectious Asia 1 Shamir and O_1_ Manisa FMDV confirms that this material is not infectious (Figure 3). These putative 75S particles derived from inactivated antigen are not recognized by M170F, recognized with reduced efficiency by M332F, and recognized well by M379F (Figure 2). Acidification of crude inactivated antigen did not fully convert the putative 75S particles of strains SAT2 SAU/2/00 and Asia 1 Shamir and the 146S particles of strain Asia 1 Shamir into 12S particles. We, therefore, recommend that preparation of a 12S standard for use in the 12S-specific M3 ELISA is preferably done by SDG purification of 146S particles and subsequent heating. The preparation of 12S standards by conversion of 146S present in crude antigen into 12S as done earlier by us (17) is inaccurate as it ignores the 12S particles already present in the sample and the 12S particles derived from 75S particles.

The ELISAs developed here for quantification of 146S particles of strains Asia 1 Shamir and SAT2 SAU/2/00 can be used for quality control of FMD vaccines during and after manufacturing. There is a need for such quality control as many locally produced FMD vaccines show poor quality (45, 46). Several other ELISAs have been described recently for this purpose (7, 12–17). There are only three reports of ELISAs specific for 146S particles (15–17). They were only suitable for O and A serotype strains. Our VHH-based ELISAs that detect 146S particles of SAT2 and Asia 1 serotype strains thus complement the currently available 146S-specific ELISAs. The serotype and strain specificity of the VHHs are advantageous for independent quantification of different FMD strains in multivalent vaccines but has the disadvantage that their use is limited to the FMD strains recognized by these VHHs. The ELISAs developed here can also be used for analysis of FMDV stability in oil-adjuvanted vaccines as described earlier for O_1_ Manisa FMDV using the M170F ELISA (17, 18). As compared to the recently described thermofluor assay for measuring FMDV stability (47) our ELISAs have the advantage of increased sensitivity but disadvantage of strain specificity.

## Ethics Statement

Llama immunizations were performed after ethical review by Wageningen Bioveterinary Research and in accordance with Dutch national guidelines on animal use.

## Author Contributions

MH and EP performed experiments. MH, JS, PE, BC, and AD conceived and designed experiments and analyzed data. MH, JS, and AD wrote the manuscript. All the authors read and critically reviewed the manuscript.

## Conflict of Interest Statement

The authors declare that the research was conducted in the absence of any commercial or financial relationships that could be construed as a potential conflict of interest. The reviewer, MG, and handling editor declared their shared affiliation, and the handling editor states that the process nevertheless met the standards of a fair and objective review.
